# Mapping functional hemodynamic and metabolic responses to dementia: a broadband spectroscopy pilot study

**DOI:** 10.1117/1.JBO.30.S2.S23910

**Published:** 2025-09-03

**Authors:** Deepshikha Acharya, Emilia Butters, Alexander Caicedo, Li Su, John O’Brien, Gemma Bale

**Affiliations:** a University of Cambridge, Electrical Division, Department of Engineering, Cambridge, United Kingdom; b University of Cambridge, School of Clinical Medicine, Department of Psychiatry, Cambridge, United Kingdom; c Pontificia Universidad Javeriana, Department of Electronic Engineering, Bogota, Colombia; d University of Sheffield, Department of Neuroscience, Sheffield, United Kingdom; e University of Cambridge, Department of Physics, Cambridge, United Kingdom

**Keywords:** broadband near-infrared spectroscopy, dementia, functional activation, metabolism, cytochrome c-oxidase

## Abstract

**Significance:**

Broadband near-infrared spectroscopy (bNIRS) can simultaneously monitor several chromophores, including the oxidative state of cytochrome c-oxidase (oxCCO), an oxygen metabolism biomarker, the activity of which is altered in Alzheimer’s disease. Being a portable and noninvasive neuromonitoring technique, bNIRS could provide accessibility to brain-specific biomarkers and aid in the dementia diagnostic pathway.

**Aim:**

We use bNIRS-recorded functional hemodynamic and oxCCO changes to assess their relevance in Alzheimer’s disease diagnosis.

**Approach:**

Using a visual stimulus paradigm, we recorded functional changes in oxy-, deoxy-hemoglobin and oxCCO in three similarly aged cohorts: healthy controls (n=5), individuals with mild cognitive impairment (n=7), and individuals with early Alzheimer’s dementia (n=7). We then selected features from these functional responses to find the best correlation with clinical cognitive markers (cognitive and behavioral test scores and clinical diagnoses) using canonical correlation analysis (CCA).

**Results:**

We found individual variations in peak amplitude and time-to-peak for all the stimulus-evoked bNIRS signals across the three cohorts. CCA showed a strong correlation between bNIRS features and the clinical cognitive markers (r=0.902). However, repeating the same analysis by excluding the bNIRS oxCCO features leads to a significantly lower correlation (r=0.687) with the clinical markers.

**Conclusions:**

oxCCO could be a crucial biomarker, partly explaining cognitive differences with dementia. bNIRS uniquely provides a portable and noninvasive technique to monitor several chromophores simultaneously, including oxCCO, with potential future applications in diagnosing and tracking dementia progression.

## 1 Introduction

Dementia arises from complex and heterogenous pathological conditions, which lead to a general decline in cognition and impairments in daily function. There is huge variability in the clinical presentation of symptoms and underlying neuro-vascular degeneration, not only in prodromal cases but also within specific dementia subtypes.^1^ An early and differential diagnosis of dementia requires a holistic understanding of the presenting symptoms and their underlying causes. A combination of neuropsychological tests and neuroimaging techniques can help provide subject-specific, comprehensive differential diagnosis. Neuropsychological tests assess several domains, such as learning, retention, working memory, and motor function, to quantify domain-specific cognitive decline.^2^ In Alzheimer’s disease (AD), studies have consistently identified impairments in executive function and working memory. These cognitive deficits are also observed in the pre-clinical stage of mild cognitive impairment (MCI).^3^ Longitudinal monitoring using neuropsychological assessments, such as the mini-mental state examination (MMSE)^4^^,^^5^ and tests evaluating executive dysfunction, can be instrumental in tracking the progression of AD. Concurrently, neuroimaging techniques such as magnetic resonance imaging (MRI) and positron emission tomography (PET) can provide structural, molecular, and some functional insights into the underlying causes of the cognitive decline.^6^

In recent years, near-infrared spectroscopy (NIRS) has emerged as an alternate neuromonitoring technique in dementia research, bringing promise with its accessibility and portability relative to conventional neuroimaging methods.^7^ NIRS uses the differential absorption of light in tissue in the near-infrared wavelengths (∼600 to 1200 nm) to quantify changes in concentration of different tissue chromophores. Most common NIRS systems use sources operating at two wavelengths and measure the light received through a photodiode. This allows NIRS to resolve for two chromophores, most commonly, oxygenated (ΔHbO) and deoxygenated (ΔHbR) hemoglobin. During functional tasks, NIRS is often used to measure stimulus-evoked hyperemia, characterized by an increase in local ΔHbO (and a decrease in ΔHbR) as oxygen is supplied to the cerebral region of activation. Previous NIRS studies have shown alteration in these functional hyperemic signals in response to sensory stimuli with MCI and AD compared with healthy controls (HCs), corroborating functional MRI findings.^7^ In addition to oxygen supply, local oxygen metabolism is a critical indicator of brain health. Metabolic dysfunction is exaggerated in AD and may signal underlying issues such as neurovascular uncoupling and mitochondrial insufficiency.^8^ Therefore, metabolic biomarkers have the potential to provide valuable insights into the pathophysiological pathways of AD.

Broadband NIRS (bNIRS) measures the differential absorption of NIR light through tissue over a range of wavelengths simultaneously. Using a broadband light source, such as a simple halogen bulb, paired with a spectrometer to detect the light received from the tissue, bNIRS can resolve for several chromophores concurrently. In addition to measuring changes in ΔHbO and ΔHbR, bNIRS can also detect concentration changes in the oxidation state of the mitochondrial enzyme cytochrome c-oxidase (CCO). Changes in the oxidation state of CCO (ΔoxCCO) represents changes in brain metabolism and has been validated in pre-clinical,^9^ clinical,^10^^,^^11^ and sensory stimulation^12^^–^^14^ research studies. Pre-clinical studies involving oxidative manipulations have also demonstrated minimal interference between bNIRS-recorded hemoglobin and oxCCO signals, indicating a degree of independence between these two measures.^15^ Although the relationship between ΔoxCCO concentration and AD pathology might not be linear, studies have shown that amyloid-β protein precursors reportedly block mitochondrial CCO, and consequently this inhibition could instigate the shift toward the amyloidogenic pathway.^16^ Post-mortem analyses have shown a 25% to 30% decrease in cortical CCO activity in AD patients, which correlated with the severity of cognitive impairment.^17^^,^^18^ Meanwhile in at-risk groups with a maternal history of AD^19^ and subjects with MCI,^20^ a decline in platelet mitochondrial CCO has been observed.

In this study, we use bNIRS to measure changes in local brain activity during visual sensory stimulation. We estimate functional changes in ΔHbO, ΔHbR, and ΔoxCCO across healthy controls (HC), individuals with MCI, and those with early AD dementia. Signal features such as peak amplitude (PA) and time-to-peak (TTP) are used to calculate ΔHbO, ΔHbR, and ΔoxCCO functional metrics. Hemoglobin difference (ΔHbD=ΔHbO−ΔHbR) is used as a proxy for cerebral blood flow.^21^ Time lag between ΔHbD and ΔoxCCO during stimulus period is used as a proxy for neurovascular coupling, where an absence of the traditional ΔHbD lagging ΔoxCCO could indicate a mismatch in oxygen supply and demand. Finally, we analyze how these bNIRS signal metrics relate to the severity of cognitive impairment, with a particular emphasis on the role of ΔoxCCO-derived measures.

## 2 Materials and Methods

### 2.1 Study Protocol

This study was conducted under the Optical Neuroimaging and Cognition protocol (ONAC; IRAS project ID: 319284) sponsored by the University of Cambridge and the Cambridgeshire and Peterborough NHS Foundation Trust. Participants expressing voluntary interest in the study who satisfied the eligibility criteria were provided with study-related documentation and an official invitation. Home visits were scheduled, if convenient, for neuropsychological assessment and subsequently bNIRS recordings. Alternatively, participants underwent bNIRS recordings on site in the Department of Psychiatry at University of Cambridge. No difference in signal quality was found between at-home and on-site recordings. Individuals with severe dementia (MMSE<12), history of traumatic brain injury, history of excessive drug or alcohol use, conditions affecting hemodynamics and metabolism, or significant physical or psychiatric illnesses were excluded from the study. Participants were classified into HCs, people with MCI, and people with diagnosed dementia due to AD. Here, MCI refers to the severity of impairment (with AD being the potential cause), and AD refers to the cause of the impairment (with dementia being the severity). Clinical groups had formal diagnosis from a memory clinic and fulfilled the diagnostic criteria for either MCI^22^ or AD.^23^

Upon consent, each participant was scheduled for an at-home neuropsychological assessment across a range of cognitive functions. For each participant, a cumulative executive function test (ExFT) score was calculated from their individual scores in test for inference sensitivity (conflicting instructions), inhibitory control (go–no go), and digit span. In addition, participants underwent the MMSE,^4^ and these scores were incorporated into the subsequent analysis. Any participant who did not complete MMSE or ExFT was not included in further analysis. ExFT was scored out of 19, and MMSE was scored out of 30. In both cases, lower scores indicated more cognitive impairment. Statistical testing for inter-group differences in the scores was done using a Wilcoxon rank sum test (*ranksum*, MATLAB 2023a). bNIRS recordings were a small pilot portion of the larger study trying to understand the role of NIRS, specifically high-density NIRS, in dementia diagnostics.

Measurements were performed using a miniature bNIRS system^24^ with the Ocean Optics HL2000 white light source and a long-pass filter with a cut-on wavelength of 630 nm (Thorlabs, Newton, New Jersey, United States, FGL630) to limit input light to the near-infrared range. A custom Wasatch spectrometer (Wasatch Photonics, USA, WP-VISNIRX-C-S-25) configured for 650 to 910 nm was used to detect the light received from the tissue. Custom 90 deg flat optical fibers (Engionic Fiber Optics GmbH, Berlin, Germany) were used to interface devices (source and detector) to the participant. An optode holder with a source detector separation of 3 cm was 3D printed from flexible resin to have a comfortable probe design and provide maximal contact to the participant head. The probe was attached to the participant head using a fabric band.

**Fig. 1:**
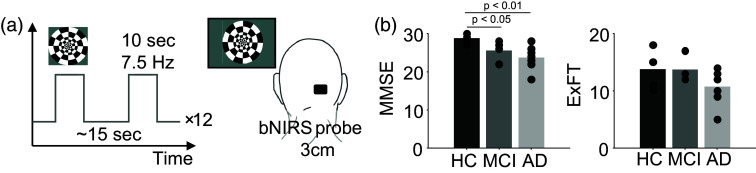
Experimental setup and cohort: (a) visual stimulus protocol and the bNIRS setup and (b) neuropsychological test scores for the three cohorts based on clinical diagnosis. Each bar shows the mean score, and the points overlaid show individual scores for each cohort. MMSE was scored out of a maximum of 30 and ExFT out of 19.

The probe was placed over the right visual cortex, while a full-field checkerboard stimulus was presented to the participant. A radial checkerboard stimulus, reversing at 7.5 Hz for 10 s was repeated 12 times with an inter-block rest period of 15 s, jittered by 0.1 s.^25^ The stimulus was implemented in Python using PsychoPy (v 2021.2.3).^26^ A schematic representation of the experiment structure is shown in Fig. 1(a).

### 2.2 Data Preprocessing

The bNIRS system recorded raw intensity spectrum once every 1.5 s (integration time) for the duration of the experiment. Initial signal quality check was performed on these raw spectra. Participant data was rejected from further analysis if the spectral intensity was too low (≤1000) or noise in the spectra made it unsuitable for further analysis. The former was a quantitative threshold, whereas the latter was through visual inspection. Examples of these cases are given in Fig. S1 in the Supplementary Material. In case of sporadic noise spikes in some spectrum, noise timepoints were identified for removal, and participant data were further analyzed. For this, at each wavelength, a timeseries of intensity was extracted from the spectra. Timepoints where the signal z-score crossed a threshold of ±5 were noted for each wavelength.

The spectral data were converted to changes in concentration (ΔHbO, ΔHbR, and ΔoxCCO) using the UCLn algorithm, a generalized modified Beer–Lambert law algorithm.^27^ Previously noted noise timepoints were removed from the concentration values, and the missing data filled in using spline interpolation. The concentration values were re-sampled to 2 Hz, and a fast-Fourier transform (FFT) was performed on each concentration value. Participants were rejected from further analysis if they lacked a prominent stimulus-locked peak in any of their concentration FFTs at the stimulus block frequency^12^ of 0.04 Hz. An example of this is shown in Fig. S1 in the Supplementary Material. Finally, a wavelet motion correction^28^ with an inter-quartile range of 1.5 (adapted from Homer3 toolbox^29^) was applied to remove motion spikes in the concentration timeseries. The resulting signals were filtered using a third-order Butterworth low-pass filter (0.08 Hz cutoff frequency) in series with a fifth-order Butterworth high-pass filter (0.01 Hz cutoff frequency) to remove low-frequency noise and physiological signals such as Mayer waves, respiration, and heart rate (*butter* and *filtfilt*, MATLAB2023a).

### 2.3 Functional bNIRS Responses and Feature Selection

Stimulus-evoked functional responses and associated features were calculated for ΔHbO, ΔHbR, and ΔoxCCO, respectively. A hemodynamic and metabolic response was expected for each block of stimulus. At the beginning of each stimulus block, a manual event marker was input by the experimenter, which was used to epoch each timeseries into stimulus-evoked responses. An epoch consisted of 10 s of stimulus presentation followed by 15 s of baseline for a total block duration of 25 s. To remove any effects of baseline drifts, an average of 3 s before stimulus onset (baseline) was subtracted from the successive epoch for each concentration signal, respectively. Furthermore, to remove epochs with no response, if the peak response was within ±3 standard deviations from the mean of the pre-stimulus period, the respective epoch was rejected. This lack of response could occur due to participant disengagement or high noise in the signal. The remaining epochs were averaged to give a single stimulus-evoked ΔHbO, ΔHbR, and ΔoxCCO response per participant.

Two features were selected to quantify these evoked responses for further analysis. The widest prominent peak from the average evoked signal was identified (*findpeaks*, MATLAB2023a) as the hemodynamic response. This gave the PA and the corresponding time was the TTP.

In addition, cross-correlation (*xcorr*, MATLAB2023a) between hemoglobin difference (ΔHbD) and ΔoxCCO timeseries was calculated spanning ±12.5  s. The lag/shift corresponding to maximum correlation was used to quantify the time lag between the two signals. A positive shift/lag indicates signal ΔHbD lagging ΔoxCCO changes, and a negative shift/lag indicates ΔHbD leading ΔoxCCO changes. Wilcoxon ranksum test (*ranksum*, MATLAB2023a) was used to test the significance between groups for all the bNIRS features.

### 2.4 Canonical Correlation Analysis

Canonical correlation analysis (CCA) is a statistical method used to assess multivariate relationships. This technique is especially useful when working with intercorrelated variables, which is often the case in complex disease classification and neuroscience.^30^ To apply CCA to our analysis, two variable sets were created—bNIRS metrics and cognitive metrics. The bNIRS metrics comprised PA and TTP for ΔHbO, ΔHbR, and ΔoxCCO each, and the time lag between ΔHbD and ΔoxCCO. Meanwhile, the cognitive metrics comprised the MMSE and ExFT scores and a cohort code based on their clinical diagnosis (1 for AD, 2 for MCI, and 3 for HC). CCA was used to identify the relationship between bNIRS and cognitive metrics and assess the amount of variance in one, explained by the other set. In addition, associated loadings to each of the variables helped gauge their role in this prediction. Nonparametric statistical testing was done by creating a null set,^30^ where cognitive metrics were randomly drawn from their set of possible values (1 to 30 for MMSE, 1 to 19 for ExFT, and 1 to 3 for the cohort code), and the resulting set was used to calculate CCA with the original bNIRS metrics. This was repeated 5000 times to create a distribution of canonical correlations with a null cognitive set. Significance was tested by comparing the original canonical correlation value with the 90th percentile of this null distribution.

To specifically test the importance of adding ΔoxCCO in this analysis, CCA was repeated, excluding the ΔoxCCO-related signal metrics (PA and TTP of ΔoxCCO and time lag). The resulting canonical correlation was then compared with the initial findings using bootstrap analysis. Bootstrapping was done by randomly drawing paired samples from the canonical variate set 5000 times, with replacement, and calculating the corresponding correlations to create a distribution of correlation values. This was done for each test case (with and without ΔoxCCO), and the resulting distribution of correlations was compared with a test for significant differences using a two-tailed t-test.

## 3 Results

Data were collected from 56 participants (HC = 23, MCI = 19, and AD = 18). After applying the data rejection criteria outlined earlier, we rejected eight participants each due to the low intensity of received light and noisy recorded spectra. In addition, 21 participants were rejected due to the lack of a reliable stimulus-locked response in the bNIRS-recorded concentration signals. The extremely high attrition rate may be attributed to factors such as poor contact of the optical probes due to the thickness, noise due to motion or ambient light, or lack of participant engagement during the passive-stimulus task. We address potential solutions in Sec. 4. Here, we report data across three similarly aged groups of HC (n=5, age: 72.72±9.57 years), MCI (n=7, age: 78.14±5.3 years), and AD (n=7, age: 78.4±7.57 years).

The average and individual scores for MMSE and ExFT across the three cohorts are plotted in Fig. 1(b). As expected, scores for both cognitive tests were the lowest for the AD group (MMSE: 23.7, ExFT: 10.7), followed by MCI (MMSE: 26.1, ExFT: 12.6) and the highest for HC (MMSE: 28.8, ExFT: 13.8). MMSE scores for MCI and AD were significantly lower than HC ($p\text{-}{\text{value}}_{\mathrm{MCI}\text{-}\mathrm{HC}}=0.02$, $p\text{-}{\text{value}}_{\mathrm{AD}\text{-}\mathrm{HC}}=0.007$). ExFT scores showed no significant group differences.

### 3.1 Functional bNIRS Responses and Feature Differences between Cohorts

**Fig. 2:**
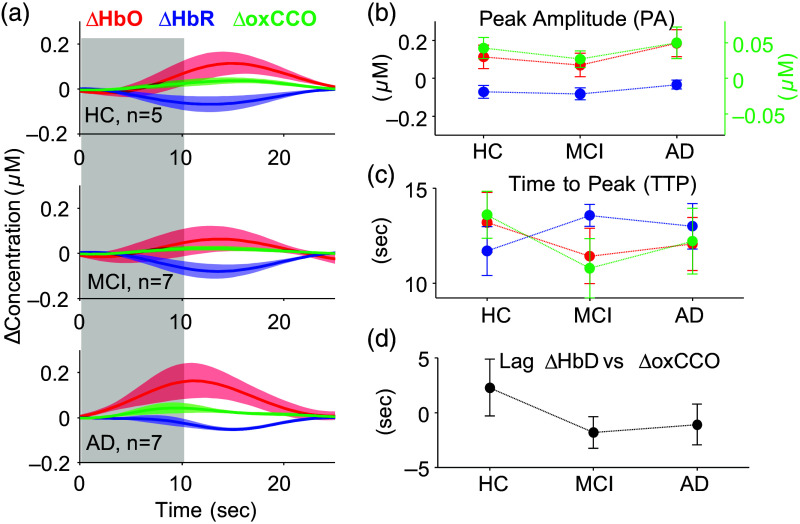
Functional responses to visual stimulation recorded by bNIRS: (a) changes in concentration of ΔHbO, ΔHbR, and ΔoxCCO epoched at the stimulus onset to 25 s [10 s stimulus block (gray) + 15 s rest]. For each group, the bold line shows the average response within the group, and the shaded region shows the standard error around the mean. (b)–(d) The PA, TTP, and lag for each group. The filled circles show the mean, and the lines show the standard error. For the entire figure, values associated with ΔHbO are in red, ΔHbR in blue, and ΔoxCCO in green.

As expected, in response to visual stimulus, we observed increases in ΔHbO and ΔoxCCO and a decrease in ΔHbR across all three cohorts. Figure 2(a) shows the average functional epochs for the different bNIRS signals for HC, MCI, and AD groups. Average participant responses were grouped based on their clinical diagnosis. Average PA for ΔHbO hemodynamic responses was the highest in the AD group (0.18±0.07  μM), followed by HC (0.11±0.06  μM), and the lowest for MCI (0.06±0.06  μM). A similar trend was observed for ΔoxCCO (HC=0.04±0.01  μM, MCI=0.03±0.01  μM, and AD=0.05±0.02  μM, respectively) and ΔHbR (HC=−0.07±0.03  μM, MCI=−0.08±0.03  μM, and AD=−0.03±0.02  μM, respectively). Furthermore, on average, an early TTP was seen in the MCI group for ΔHbO (11.4±1.45  s) and ΔoxCCO (10.7±1.55  s) responses compared with AD (ΔHbO=12.1±1.39  s, ΔoxCCO=12.2±1.73  s) and HC (ΔHbO=13.2±1.59  s, ΔoxCCO=13.6±1.23  s). The opposite trend was observed for ΔHbR with TTP on-average being earlier for HC (11.7±1.28  s) compared with MCI (13.6±0.58  s) and AD (12.2±1.19  s). Finally, ΔHbD was leading ΔoxCCO for MCI (1.7±1.44  s) and AD (1.07±1.87  s) but lagging ΔoxCCO for HC (2.3±2.6  s). Figures 2(b)–2(d) show the average ± standard error for PA, TTP, and lag, respectively, for each group. It must be noted here that all values are reported as mean ± standard error, and none of the metrics showed significant difference between groups.

### 3.2 bNIRS and Cognitive Metric Correlations

**Fig. 3:**
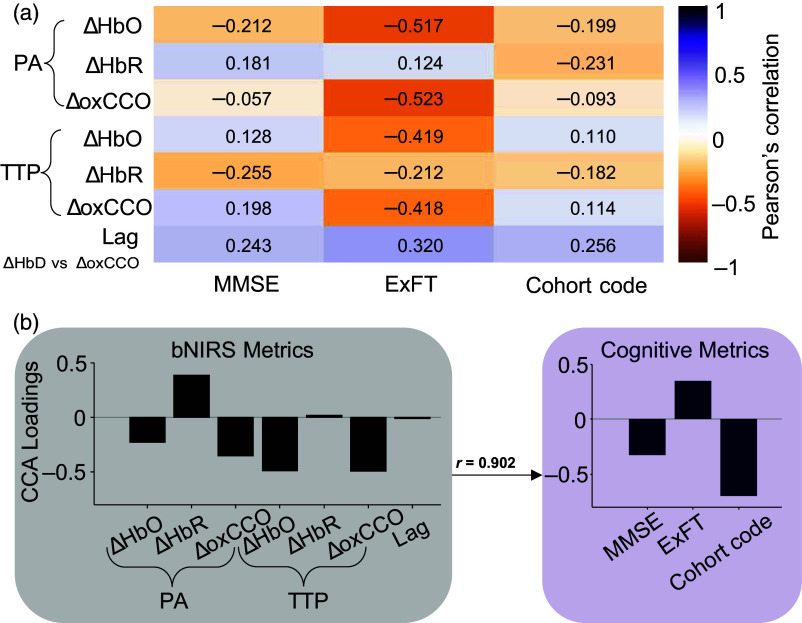
Correlations between bNIRS and cognitive metrics: (a) Pearson’s correlation between the two sets of metrics. (b) CCA results between the two sets of metrics—the bar graphs represent the corresponding loadings for each variable. All the bNIRS metrics are shown in black, and the cognitive metrics are shown in purple. The two sets were found to have a canonical correlation of 0.902. This value was found to be greater than the 90th percentile of the null distribution of canonical correlations.

A Pearson’s correlation was calculated between the bNIRS metrics (PA and TTP for ΔHbO, ΔHbR, and ΔoxCCO and time lag) and the cognitive metrics (MMSE and ExFT scores and cohort code) to assess how they each contributed to explaining the variance in the other metric. Data from all participants were pooled for this analysis. ExFT scores showed the strongest correlation with all the bNIRS metrics, specifically PA and TTP for ΔHbO and ΔoxCCO. Figure 3(a) shows the correlation between each set of variables.

The same pooled dataset across all participants from above was used to perform CCA between the bNIRS and the cognitive metrics. The highest correlation corresponding to the first component of the bNIRS and cognitive metrics was 0.902. Loadings were also calculated from the first components to understand their relationship to the corresponding metrics. Figure 3(b) shows the loadings for each metric and the highest canonical correlation between the two variable sets.

### 3.3 Contribution of ΔoxCCO in Clinical Metric Correlations

**Fig. 4:**
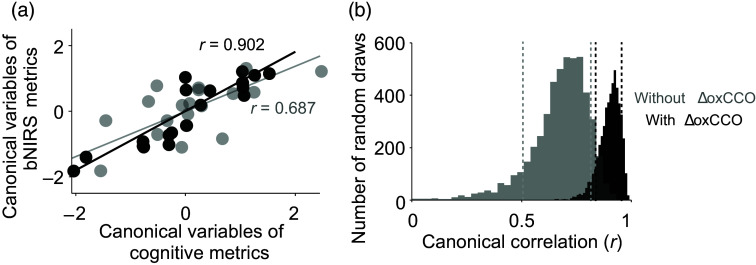
CCA for two cases—bNIRS metrics with ΔoxCCO (black) and bNIRS metrics without ΔoxCCO (gray): (a) the CCA correlation between the first component of the canonical variables for the two cases. This shows the maximum possible correlation between the linear combination of the two metrics (canonical variables).^30^ (b) The bootstrap distribution of correlation values for the two cases. The dashed vertical lines show the 10th and 90th percentiles of the respective distributions.

Canonical loadings for ΔoxCCO PA (−0.35) and TTP (−0.49) were found to be among the highest for the bNIRS metrics, though the same for time lag was amongst the lowest (−0.01). We further tested the importance of ΔoxCCO in accounting for the variance in the clinical data by performing CCA once including ΔoxCCO and once without (as would be the case with traditional NIRS). When excluding ΔoxCCO, the new bNIRS metric set only used PA and TTP for ΔHbO and ΔHbR. Figure 4(a) shows the correlations between the first components for each of the test case—with ΔoxCCO (r=0.902) and without ΔoxCCO (r=0.687). To test the significance of the difference in the correlations, we also performed a bootstrap analysis followed by a two-tailed t-test. Figure 4(b) shows the distribution and the 10th and 90th percentiles of each distribution. The CCA correlation when using ΔoxCCO was found to be significantly higher compared to without ΔoxCCO (p-value≪0.05).

## 4 Discussion

In this work, we propose the use of bNIRS for the first time in dementia research, leveraging its ability to resolve multiple biomarkers simultaneously. We specifically focus on the role of ΔoxCCO, a key mitochondrial enzyme, in AD. Using bNIRS in a visual stimulus paradigm, we estimate visual-evoked hemodynamic (ΔHbO and ΔHbR) and metabolic (ΔoxCCO) changes across three similarly aged cohorts—AD (n=7), MCI (n=7) and HC (n=5). To comprehensively understand the relationship between the recorded signals and diagnosis, we distilled the results in two variable sets, bNIRS metrics (PA, TTP, and time lag) and cognitive metrics (MMSE and ExFT scores and the cohort code), and used CCA to find the correlation (r=0.902). Applying the same canonical analysis without the ΔoxCCO-derived metrics yielded in a significantly lower (p-value≪0.05) canonical correlation (r=0.687). This section discusses our results in the context of existing literature and suggests improvements for the expansion of this work.

We observed an expected decrease in average neuropsychological test scores [Fig. 1(b)] from HC to AD, for both MMSE and ExFT. MMSE test scores were significantly lower (p-value≪0.05) for MCI and AD compared with HC. Meanwhile ExFT scores showed no significant difference between groups. The variance in individual scores was higher within groups for ExFT compared with MMSE. Previous work has linked this heterogeneity in executive dysfunction in mild AD to potential differences in underlying disease processes and secondary conditions.^31^ Figure 1(b) shows individual scores overlaid on the corresponding bar plot showing group averages.

During functional bNIRS recordings, we also observed expected functional hyperemic responses, though their PA and TTP varied across the three groups (Fig. 2). Neurovascular dysfunction has been widely reported with cognitive impairment, corroborated by the hypoactivation reported during sensory stimulation/tasks with increasing cognitive decline.^32^ Consequently, we did observe a decrease in PA and a faster TTP in MCI compared with HC for ΔHbO. This reduction in occipital activation has been previously reported during a working-memory task^33^ in MCI compared with HC groups. Furthermore, Bu et al.^34^ also showed a reduction in coupling strength at resting state across different brain regions, including the occipital lobe, in MCI compared with HC. Although the MCI group showed reduced activation amplitude compared with HC, we observed the opposite for the AD group. An average increase in PA was seen between HC and AD for ΔHbO. This hyperactivation in the visual cortex in early-stage AD has been seen in previous studies and linked to compensatory mechanisms or dysregulation of the neurovascular homeostatic cycle.^35^^,^^36^ This could also explain the faster TTP in MCI and AD compared with HC for ΔHbO [Fig. 2(c)]. Interestingly, the difference in TTP between ΔHbO and ΔHbR was the highest for MCI, which could originate from systemic sources or cortical hemodynamic differences^37^ amplified by the neuro-vascular deficiencies with dementia. This difference in TTP was, however, absent for AD. Further investigation by potentially isolating systemic effects with short channel regression might shed light on differences in TTP between the hemodynamic signals with dementia. We also observed a decrease in ΔHbR PA response from HC to AD despite an increase in ΔHbO PA. Metabolic differences with AD could partially explain this discrepancy.

CCO is an important part of the oxidative metabolism of glucose, featuring in the electron transport chain. Changes in absolute concentrations of complex IV (CCO) have been related to AD. In a post-mortem study relating CCO changes with AD, they found the expected decrease in CCO in the frontal and parietal regions; however, there was a nonsignificant elevation in CCO in the occipital cortex.^38^ Contrarily, another study^17^ found a 25% to 30% decrease in CCO across all cortical regions studied, including the occipital cortex. Beyond absolute CCO concentration, in AD mouse models, a deficient oxygen consumption at complex IV in the mitochondria has been observed^39^^,^^40^ and could specifically explain reduced concentrations of oxCCO. Although most measurements of CCO are post-mortem in the cases of dementia, measurements of glucose metabolism with FDG-PET have enabled similar assessment of regional cortical metabolic deficiencies during disease progression. Condition-specific hypometabolism have been reported in AD^41^ and mapped to eventual disease manifestation in MCI.^42^ During functional tasks, a study found reductions in cerebral metabolic rate of glucose in AD compared with HC during a visual recognition task using FDG-PET.^43^ We also observed a small decrease in functional ΔoxCCO PA in the visual cortex for MCI group compared with HC (Fig. 2). However, ΔoxCCO PA was highest for AD, potentially indicating hypermetabolism. Another common measure of metabolism in MRI studies is cerebral metabolic rate of oxygen consumption (${\mathrm{CMRO}}_{2}$). A study showed reduction in frontal cortical blood flow and ${\mathrm{CMRO}}_{2}$ as well as their correlation (coupling) in participants with subjective cognitive decline compared with controls.^44^ Aside from correlation across a series of conditions/tasks/brain regions, coupling can also be shown using the time delay between blood flow (oxygen supply) and metabolism (oxygen demand). We found oxygen demand (ΔoxCCO) to lead oxygen supply (ΔHbD) in HC by 2.3±2.6  s, which is expected during functional activation in a healthy brain.^45^ However, this was reversed in MCI and AD where ΔHbD was found to lead ΔoxCCO. No differences were observed in the correlation values corresponding to these lag times. The delayed metabolic response compared with flow and the faster TTP of ΔoxCCO and ΔHbO in MCI and AD could indicate underlying neuronal deficits, impaired cerebrovascular reactivity, or potential neurovascular uncoupling. It must also be noted here that ΔoxCCO may not match ${\mathrm{CMRO}}_{2}$ under some conditions,^46^^–^^48^ which might affect the interpretation of our results against the few existing functional activation dementia literature that usually use ${\mathrm{CMRO}}_{2}$. AD has been dubbed as a metabolic disease^49^ with markers such as oxCCO potentially being key to aiding diagnosis. Our work here is the first to bring the more established mitochondrial marker (oxCCO) for AD to noninvasive functional studies. Further investigation of these results with different sensory tasks, a better measure of local blood flow, and simultaneous assessment of ${\mathrm{CMRO}}_{2}$ would help understand the results in the context of the existing literature. Finally, although there were trends between groups for the bNIRS metrics, none of the trends were found significant. This could be due to the small sample size or high inter-subject variability in prodromal and early-AD groups arising from heterogeneity in underlying disease pathways and differences in disease progression.^1^ All the collected data were pooled into two variable sets—bNIRS metrics and cognitive metrics—and a pairwise Pearson’s correlation was calculated. The strongest correlation was found between the ΔHbO and ΔoxCCO PA and TTP and the ExFT [Fig. 3(a)]. Recently, more studies have indicated early impairment of executive function in AD, possibly arising from the degeneration of prefrontal cortex.^50^ However, given the interdependence of variables in each set, conclusions from pairwise Pearson’s correlation might be conflated. For a more comprehensive understanding, we used CCA, a multivariate statistical analysis method. With CCA, we found that the first component of bNIRS metrics accounted for over 80% of the variance in the first component of the cognitive metrics [Fig. 3(b)] with a correlation of r=0.902. This correlation was found to be greater than the 90th percentile of the null canonical correlation distribution created by shuffling a randomly drawn set of cognitive metrics. However, the canonical correlation significantly decreased (p-value≪0.05) to r=0.687 when not using ΔoxCCO-derived metrics, now accounting for only 50% of the variance in cognitive metrics (Fig. 4). This highlights the crucial role bNIRS-recorded ΔoxCCO could play in understanding differences in cognitive impairment with AD. This was supported by the correspondingly high loadings for the ΔoxCCO features, particularly PA and TTP, which represent its high contribution to the final correlation value [Fig. 3(b)]. We also observed a similarly high contribution from ΔHbO and an expected inverse contribution from ΔHbR given its negative magnitude for PA and opposite trend in TTP compared with ΔHbO and ΔoxCCO [Fig. 3(b)]. Overall, large loadings of the cognitive metrics indicate the role of all of them—MMSE, ExFT, and cohort code based on diagnosis, in the relationship with bNIRS metrics. Finally, our CCA results indicate the importance of using multiple biomarkers, especially metabolic markers such as ΔoxCCO to improve differential diagnosis.

Crucially, it must be noted that the functional bNIRS data were collected at-home or at a controlled-environment study site, based on participant convenience. No data quality differences were found between the two study locations. Our work is among the first to use bNIRS for AD, especially at-home. This highlights the importance of portable and noninvasive neuromonitoring techniques such as bNIRS in enabling at-home monitoring of symptoms and aiding dementia diagnosis.

The results here are from a small cohort of participants using a single channel bNIRS setup during a visual task. Due to challenges in data collection, despite the 56 complete datasets of recorded participants, only 19 were used for final analysis. This led to three cohorts with small sample sizes that were not perfectly age- or sex-matched. The influence of age and sex on vasculature could impact the final results. Future improvements to this work would include a greater number of bNIRS channels to have a better coverage of the sensory region, improved signal-to-noise ratio, and removal of the influences from superficial layers such as scalp. In addition, a high-density multichannel setup could enable cortical source localization,^51^ which concurrently with patient-specific MRI, could help account for signal difference due to brain atrophy, especially in advanced AD cases. Another aspect of study improvement could be in the task itself. Visual evoked responses to passive stimuli are well established in neurophysiology and specifically functional NIRS.^12^^,^^52^ These have also been previously used to note differences between HC and AD pathology.^53^^,^^54^ However, given the differences in cortical vulnerabilities with AD,^55^ a battery of functional tests probing different cortical regions could improve dementia/severity classification as well as participant engagement. Correspondingly, the bNIRS probe setup could be moved to different regions to acquire region-specific hemodynamic responses to the task. Although conventional CCA can be very informative for small datasets, it is also heavily affected by outliers, which made signal quality checks even more imperative for our analysis. Effect of outliers can be limited with better signal quality, for which we have discussed recommendations above, or with larger datasets. With larger datasets, it will be feasible to develop a classifier using machine learning algorithms and provide a more robust understanding of the role of ΔoxCCO in aiding AD diagnosis.

## 5 Conclusion

Neuropsychological assessment in combination with neuromonitoring techniques could provide early and differential diagnosis of dementia and enable targeted symptom management. Metabolic dysfunction, specifically oxCCO, could play a key role in understanding early cognitive impairment arising from AD. This work is the first to noninvasively measure oxCCO during functional tasks, at-home, with a noninvasive and wearable system (bNIRS) in a dementia population and show its utility in diagnostics. Here, we used bNIRS to measure functional responses in ΔHbO, ΔHbR, and ΔoxCCO during visual stimulation in participants with MCI, AD, and HCs. CCA revealed a strong link between bNIRS signals and cognitive scores, which significantly weakened when ΔoxCCO metrics were excluded. Metabolic measurements from bNIRS (ΔoxCCO) show promise for dementia diagnosis, treatment monitoring, and accessible at-home quantitative assessment. This study provides a method and initial evidence supporting bNIRS use in dementia research.

## Supplementary Material

10.1117/1.JBO.30.S2.S23910.s01

## Data Availability

Relevant code for the analysis presented in this paper are available through FigShare under 10.6084/m9.figshare.29222378
