# Supplementary material for: Mapping functional hemodynamic and metabolic responses to dementia: a broadband spectroscopy pilot study
Source: J Biomed Opt. 2025 Sep 3;30(Suppl 2):S23910. doi: 10.1117/1.JBO.30.S2.S23910 (PMC12407020; doi:10.1117/1.JBO.30.S2.S23910)
Supplement: Supplementary file 1 [file JBO_030_S23910_SD001.pdf]

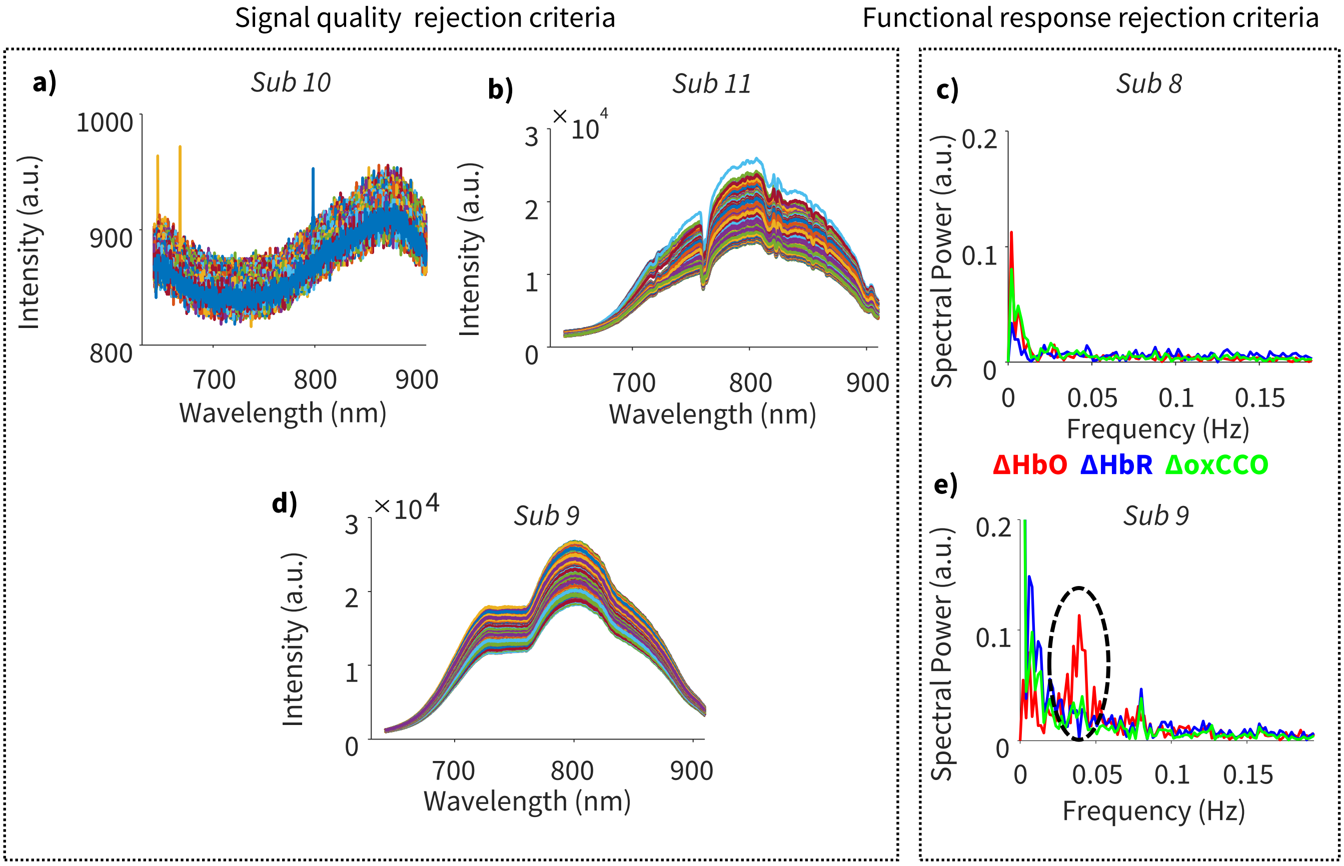

Supplementary figure S1 – Rejection criteria based on signal quality of the recorded spectra. a) shows an example of rejection based on spectral intensity ≤ 1000 which was considered too low to derive meaningful information. b) shows an example of rejection due to noisy spectra where contamination from ambient light or improper contact (to participant visual cortex), among other reasons, made the spectra too noisy to meaningfully process. d) shows an example of spectra that was used for further processing. Spectrum at each time point for the experiment duration are overlaid in each figure.

Rejection criteria based on quality of functional response. Subject's data was rejected if no stimulus-locked (~0.04Hz) distinct peak was seen in the fast-Fourier transform of the concentration signals. c) shows an example of rejection due to the lack of a stimulus-locked peak in signal power. e) shows an example of stimulus-locked peak in at least one of the concentration signals (ΔHbO, highlighted dashed ellipse).

All the subjects selected in this figure were clinically diagnosed with AD.
